# Supplementary material for: Endemic and zoonotic cycles of cutaneous leishmaniasis depend on vector feeding preferences: An epidemiological model for Southeastern Mexico
Source: PLoS Negl Trop Dis. 2025 Dec 31;19(12):e0013786. doi: 10.1371/journal.pntd.0013786 (PMC12755797; doi:10.1371/journal.pntd.0013786)
Supplement: Attachment — Submitted filename: Review report PNTD.pdf [file pntd.0013786.s004.pdf]

Report on the manuscript (PNTD-D-25-00950) entitled "**Endemic and Zoonotic cycles of cutaneous leishmaniashis depend on vector feeding preferences: an epidemiological model for Southeastern Maxico**" by Martin et al.

The manuscript mentions a mathematical model designed to examine multiple host choices (rodents and humans) in relation to two sandfly vectors (L. cruciata and B. olmeca) in the context of Cutaneous leishmaniasis transmission. The findings indicate that the B. olmeca species primarily contributes to disease transmission among rodent populations, while the L. cruciata species mainly influences human infections. This insight is valuable for understanding targeted interventions to control Cutaneous leishmaniasis in the region. I believe the manuscript has notable merit and aligns with the aims and scope of the journal PLOS Neglected Tropical Diseases. However, several changes are necessary, and I would like to offer the following suggestions for the authors:

1. In Equation (1b), on page 7, lines 157-159, could you clarify the meaning of the variables, $V_{i,N}$, R_N, and H_N ? I could not find any information regarding these variables.

2. In the model assumptions, it states that vectors can only become infected by biting infected rodents. Therefore, the transmission terms in Equations (1a) and (1b) may be incorrect, as the human population does not contribute to vector infection.

3. In Table 1, what is the rationale for setting the initial value of the rodent population at 1,400? Additionally, why is the initial value for the human population set within the range of 60-600? Please provide epidemiological evidence to support this choice.

4. On page 11, line 230, the entry in the equation is not clearly visible.

5. On page 10, line 215, could you clarify what you mean by "mates in quadrats of 1 ha"?

6. On page 12, the authors conduct a local sensitivity analysis of parameters. However, to obtain more robust results, it is suggested that the authors perform global sensitivity analysis techniques, such as PRCC, EFAST, or Sobol.

7. On page 15, lines 337-338, the brackets are not completed.

8. On page 16, line 350, there is an extra set of parentheses that need to be addressed.

9. On page 17, lines 367-368, could you please provide the reference(s) for the claims made?

10. In Figure 2, each axis should be correctly positioned. It is difficult to understand this picture.

11. No figures are clearly visible; the authors should provide high-resolution images.

Thank you for your attention to these suggestions.
