# Supplementary material for: Endemic and zoonotic cycles of cutaneous leishmaniasis depend on vector feeding preferences: An epidemiological model for Southeastern Mexico
Source: PLoS Negl Trop Dis. 2025 Dec 31;19(12):e0013786. doi: 10.1371/journal.pntd.0013786 (PMC12755797; doi:10.1371/journal.pntd.0013786)
Supplement: Attachment — Submitted filename: Rebuttal.docx [file pntd.0013786.s005.docx]

# Response to Review Comments

Dear Editor.

We find that the review comments are extremely useful and agree that all issued raised should be corrected should it be published in PLOS Neglected Tropical Diseases. We thank you for the opportunity to revise our submission, and below are our responses to all comments. For guidance, review comments are in grey colour, and reponses are in normal black colour preceded by “R:”, placed after each comment.

## Editor

Dear Dr. Montes de Oca-Aguilar,

Thank you for submitting your manuscript to PLOS Neglected Tropical Diseases. After careful consideration, we feel that it has merit but does not fully meet PLOS Neglected Tropical Diseases's publication criteria as it currently stands. Therefore, we invite you to submit a revised version of the manuscript that addresses the points raised during the review process.

Please submit your revised manuscript within 60 days Oct 11 2025 11:59PM. If you will need more time than this to complete your revisions, please reply to this message or contact the journal office at plosntds@plos.org. When you're ready to submit your revision, log on to https://www.editorialmanager.com/pntd/ and select the 'Submissions Needing Revision' folder to locate your manuscript file.

Please include the following items when submitting your revised manuscript:

* A rebuttal letter that responds to each point raised by the editor and reviewer(s). You should upload this letter as a separate file labeled 'Response to Reviewers'. This file does not need to include responses to any formatting updates and technical items listed in the 'Journal Requirements' section below.

* A marked-up copy of your manuscript that highlights changes made to the original version. You should upload this as a separate file labeled 'Revised Manuscript with Track Changes'.

* An unmarked version of your revised paper without tracked changes. You should upload this as a separate file labeled 'Manuscript'.

## Review #1

Methods should be more detailed and aligned with the objectives of this work. The model must be adjusted according to the suggestions in the reviewer attachment. Additionally, the parameters used for this simulation should have appropriate references included.

R: Done, thank you for your comments. With regards to the references, these were included in Table 2 (lines 204-214).

The manuscript mentions a mathematical model designed to examine multiple host choices (rodents and humans) in relation to two sandfly vectors (*L. cruciata* and *B. olmeca*) in the context of Cutaneous leishmaniasis transmission. The findings indicate that the *B. olmeca* species primarily contributes to disease transmission among rodent populations, while the *L. cruciata* species mainly influences human infections. This insight is valuable for understanding targeted interventions to control Cutaneous leishmaniasis in the region. I believe the manuscript has notable merit and aligns with the aims and scope of the journal PLOS Neglected Tropical Diseases. However, several changes are necessary, and I would like to offer the following suggestions for the authors:

R: Thank you, below we report the changes made on the manuscript.

1. In Equation (1b), on page 7, lines 157-159, could you clarify the meaning of the variables, 𝑉_i, N_, 𝑅_N_ , and 𝐻_N_ ? I could not find any information regarding these variables.

R: *V_iN,_* *R_N_* and *H_N_*, are the total population sizes of vectors, rodents and humans. We have added the description in line 157 of the document with track changes.

2. In the model assumptions, it states that vectors can only become infected by biting infected rodents. Therefore, the transmission terms in Equations (1a) and (1b) may be incorrect, as the human population does not contribute to vector infection.

R: In these two equations the term in the numerator is the size of the infected rodent population; hence, the only one contributing to infection, the denominator containing *R_N_* and *H_N_* are multiplied by the frequency with which vectors feed on either species to adequately represent the probability of feeing on an infectious host among the meals in each time step. To summarize, humans do not transmit *Leishmania sp*. to vectors, but do affect the probability of finding an infectious meal.

3. In Table 1, what is the rationale for setting the initial value of the rodent population at 1,400?

R: According to rodent population density data, these fluctuate between 10-200 individuals per hectare, but actual figures change between seasons. Based on the size of the study area of ~ 125 ha (X’pujil, Campeche, see below), by extension, the minimum population size would be 1250 individuals. Therefore, the initial value of 1500 individuals roughly represents the state of the populations at the beginning of the leishmaniasis transmission season. To support this, we have included the data in the form of population estimates timelines.

Additionally, why is the initial value for the human population set within the range of 60-600? Please provide epidemiological evidence to support this choice.

R: To date there is no epidemiological basis for these susceptible population sizes. As mentioned in the methods (line 246 of the original submission), these were based on the population estimates of Xpujil, Campeche, of approximately 6000 people according to the WorldPop database (Tatem et al. 2017). Then, for the simulations we assumed that 1, 5 and 10% of the population was exposed to CL vectors via occupational risk factors. These percentages were taken from the bureau of statistics for the study region (INEGI 2024).

4. On page 11, line 230, the entry in the equation is not clearly visible.

R: This too was an artefact generated by the editorial manager; we apologise for not having verified this beforehand. We have introduced the equation as regular text as it does not require any special formats.

5. On page 10, line 215, could you clarify what you mean by "mates in quadrats of 1 ha"?

R: The actual phrasing is “... *data consists of a time-series of population density* ***estimates in quadrats of 1 ha***...” (one hectare).

6. On page 12, the authors conduct a local sensitivity analysis of parameters. However, to obtain more robust results, it is suggested that the authors perform global sensitivity analysis techniques, such as PRCC, EFAST, or Sobol.

R: Done, we have performed a global sensitivity analysis using Sobol. Method details appear in lines 262-266 and results in lines 296-303.

7. On page 15, lines 337-338, the brackets are not completed.

R: Done.

8. On page 16, line 350, there is an extra set of parentheses that need to be addressed.

R: Done.

9. On page 17, lines 367-368, could you please provide the reference(s) for the claims made?

R: These are inferences we make based on our modelling results. We have rephased to emphasise this fact:

“... *consequences. For instance, we infer that on the one hand, specialist vectors like Bi. olmeca act as efficient* ...”

10. In Figure 2, each axis should be correctly positioned. It is difficult to understand this picture.

R: We are very confused by this comment, as all axes are perfectly aligned in our submitted figures. Regardless, this figure was replaced by the global sensitivity analysis.

11. No figures are clearly visible; the authors should provide high-resolution images.

R: We apologise for this inconvenience. Although we did submit hi-res images, this was lowered by the editorial manager. We verified that the original submitted files were accessible through the links at the top of each page showing the submitted figures. Should this problem persist, please follow the links with the text “*Click here to access/download;Figure;Fig-X.png*” in the top right corner.

Thank you for your attention to these suggestions.

R: Thank you.

## Review #2

This manuscript develops a compartmental model for cutaneous leishmaniasis (CL) in the Yucatán Peninsula with two vector species (*Lutzomyia cruciata* and *Bichromomyia olmeca*), a rodent reservoir, and humans as incidental hosts. Vectors and rodents follow SI dynamics; humans follow SEIS dynamics. The authors explore scenarios and conclude that *Bi. olmeca* maintains infection in reservoirs while *Lu. cruciata* drives zoonotic spillover to humans. Going through the manuscript, I have several comments and suggestions.

R: Thank you, we hope to have adequately addressed them all.

1. In Table 2, the vector-to-human and vector-to-rodent transmission rates are defined as β_{H,i} = n_i * ln(1 − ε) and β_R = n_i * ln(1 − ε). As written, ln(1 − ε) ≤ 0, making β negative. Please verify and correct throughout (including any code/simulations), and update results if necessary.

R: Thank you, the correct form is: – *n_i_* log(1 - *ε*).

2. β_R is built from the average number of bites on humans (n_i) rather than bites on rodents (which should depend on host availability and p_i). Please explain the motivation behind this assumption in more detail.

R: The transmission rate from vectors to reservoirs, *β_R_*, has the same data deficiencies as *β_V,I_*; however, we decided to use as much information on the local epidemiology as possible; hence, we built it under the assumption that the main difference between *β_R_* and *β_H,i_* is related to the frequency of phlebotomine species bites on either species. To make this clear we added the following (lines 237-239):

“... . For *β_R_* **, to use as much data from the study area,** we used **the same** values derived for humans (Ravinovich and Feliciangeli, 2004) **but weighted by the proportion of bites on rodents. Also, i**n light of ...”

3. The model considered rodents as a reservoir; however, dogs can also act as reservoir (<https://doi.org/10.3390/tropicalmed3020043>). Please give supporting statements to discard all other reservoirs other than rodents.

R: This is correct; there is evidence of infection in dogs, but their role as reservoirs is still unclear, unlike South American leishmaniasis, the region analysed in the cited paper. Therefore, we decided to only include rodents in the model because these are the only species for which there is evidence that they act as reservoirs (Discussion lines 340-360).

4. The model assumes infectious humans cannot infect susceptible vectors. Please (a) justify the zero-infectivity assumption with citations specific to *Le. mexicana* in the study area, or (b) add a sensitivity/scenario where humans have low but non-zero infectivity to vectors.

R: The most up to date knowledge of the role of humans on *Leishmania mexicana* life cycle in the Yucatan Peninsula is contained in

- Andrade-Narvaez, F. J., Van Wynsberghe, N. R., Sosa-Bibiano, E. I., & Loria-Cervera, E. N. (2017). Eco-epidemiological and immunological features of localized cutaneous Leishmaniasis in Southeastern Mexico: thirty years of study. *The Epidemiology and Ecology of Leishmaniasis.*

where it is shown that there is no evidence that infected humans can transmit the parasite to vectors. Therefore, the assumption of zero infectivity from humans is consistent with:

- WHO (2010) Control of the Leishmaniasis: Report of the WHO Expert Committee on the Control of Leishmaniases. Geneva, 22–26 March 2010. WHO Technical Report Series 949; Bray RS (1989) Zoonoses and leishmaniasis. In DT Hart (ed.), Leishmaniasis: the Current Status and New Strategies for Control, Plenum Press, New York 171: 57–60)

where *Leishmania mexicana* transmission in the Yucatan peninsula is considered strictly enzootic. We have added both references to justify the assumption of zero-infectivity in the methods section (lines 144-145).

5. The Introduction and prior work emphasize seasonal transmission in the region, but the model lacks seasonal forcing (<https://journals.plos.org/plosntds/article?id=10.1371/journal.pntd.0003283>). Please justify this assumption.

R: This aspect of the leishmaniasis transmission system is in fact mentioned in the introduction (line 101). However, many epidemiological aspects are unresolved, unlike South America where the cited paper is based. Therefore, we only analysed transmission dynamics during the five-month season (~150 days, the length of all simulations). We have now clearly stated this in the methods (lines 247-248):

“... possible outcomes **during one leishmaniasis transmission season of five months duration (~150 days),** in response to ...”

6. Simulations are qualitative. For policy relevance, please compare predicted prevalence or incidence trajectories to data from Xpujil or nearby foci (even if approximate).

R: The most reliable and up to date figures appear in the article:

- Andrade-Narvaez, F. J., Simmonds-Diaz, E., Rico-Aguilar, S., Andrade-Narvaez, M., Palomo-Cetina, A., Canto-Lara, S. B., ... & Albertos-Alpuche, N. (1990). Incidence of localized cutaneous leishmaniasis (chiclero's ulcer) in Mexico. *Transactions of the Royal Society of Tropical Medicine and Hygiene*, *84*(2), 219-220.

Since this article was published, the social, demographic and ecological contexts have changed dramatically; hence, we consider that using its estimates as reference is misleading, although in the long term the prevalence figures produced by our model (~45%) are remarkably similar to the average reported in the referred study (~43%). In light of these considerations, we have included the long-term simulation results in the supplementary materials and further emphasised the need for improved reporting and incidence/prevalence estimates in the discussion (lines 375-384).

7. Other minor comments:

a. “sensible to transmission rates” → “sensitive to transmission rates” (Abstract/Results).

R: Done.

b. “hypothesis that” → “hypothesize that” (Methods).

R: Done.

c. Species names: ensure consistent, correct spelling and italics: Peromyscus yucatanicus (not “Peromiscus”), Ototylomys phyllotis, Heteromys gaumeri. I noticed inconsistencies in Table 1 species list.

R: Done.

d. Use either Bichromomyia olmeca or B. olmeca olmeca consistently.

R: Done.

e. Units: make p_i unit-free; confirm units for n_i and βs

R: Done.

f. Line 230, the equation is not readable.

R: Done.

## References

Andrade-Narvaez, F. J., Simmonds-Diaz, E., Rico-Aguilar, S., Andrade-Narvaez, M., Palomo-Cetina, A., Canto-Lara, S. B., ... & Albertos-Alpuche, N. (1990). Incidence of localized cutaneous leishmaniasis (chiclero's ulcer) in Mexico. *Transactions of the Royal Society of Tropical Medicine and Hygiene*, *84*(2), 219-220.

Andrade-Narvaez, F. J., Van Wynsberghe, N. R., Sosa-Bibiano, E. I., & Loria-Cervera, E. N. (2017). Eco-epidemiological and immunological features of localized cutaneous Leishmaniasis in Southeastern Mexico: thirty years of study. *The Epidemiology and Ecology of Leishmaniasis.*

Lloyd, C. T., Sorichetta, A., & Tatem, A. J. (2017). High resolution global gridded data for use in population studies. *Scientific data*, *4*(1), 1-17.

WHO Expert Committee on the Control of the Leishmaniases. Meeting, & World Health Organization. (2010). *Control of the leishmaniases: report of a meeting of the WHO Expert Committee on the Control of Leishmaniases, Geneva, 22-26 March 2010* (No. 949). World Health Organization.
